# LncRNA SOCS2-AS1 inhibits progression and metastasis of colorectal cancer through stabilizing SOCS2 and sponging miR-1264

**DOI:** 10.18632/aging.103276

**Published:** 2020-05-21

**Authors:** Zhihai Zheng, Xiaoxiao Li, Heyi You, Xiaofeng Zheng, Xiaojiao Ruan

**Affiliations:** 1 Department of Colorectal and Anal Surgery, The First Affiliated Hospital of Wenzhou Medical University, Wenzhou 325000, China; 2 Department of Vascular Surgery, The First Affiliated Hospital of Wenzhou Medical University, Wenzhou 325000, China; 3 Department of Gastrointestinal Surgery, The First Affiliated Hospital of Wenzhou Medical University, Wenzhou 325000, China

**Keywords:** colorectal cancer, SOCS2-AS1, miR-1264, SOCS2, progression

## Abstract

Abnormal expression of long noncoding RNA (lncRNA) is involved in human cancers, including colorectal cancer (CRC). However, their functional mechanism is largely unknown. In this study, we explored the roles of lncRNA SOCS2-AS1 in modulating CRC progression. We showed that SOCS2-AS1 was lowly expressed in CRC tissues and cells. SOCS2-AS1 downregulation predicted a poor prognosis in patients with CRC. SOCS2-AS1 overexpression significantly suppressed CRC cell proliferation, colony formation, EdU incorporation, cell-cycle, migration and invasion *in vitro* while SOCS2-AS1 knockdown led to an opposite phenotype. SOCS2-AS1 overexpression inhibited CRC growth and metastasis *in vivo*. Mechanistically, we discovered that SOCS2-AS1 was positively correlated with SOCS2 expression in CRC tissues. SOCS2-AS1 contributes to SOCS2 expression through restraining miR-1264. Additionally, we showed that SOCS2 silencing abrogated the suppressive effects of SOCS2-AS1 overexpression. Taken together, our results identified a novel regulatory loop in which SOCS2-AS1/miR-1264/SOCS2 axis suppresses CRC progression.

## INTRODUCTION

As the fourth most prevalent cancer, colorectal cancer (CRC) leads to a large number of cancer-associated deaths around the world [1]. Most patients are diagnosed with CRC at advanced stages due to lack of an effective diagnostic biomarker for timely screening [2]. Cancer progression and metastasis have been the major factors of CRC-induced deaths. Although some improvement has been made on the therapeutic methods, such as surgery, radiotherapy, chemotherapy and immunotherapy, the prognosis of CRC patients is far from satisfactory [3]. Therefore, it is urgently needed to understand the molecular mechanism underlying CRC progression.

In the past decades, it has been identified that noncoding RNAs (ncRNAs) are produced by the vast majority of mammalian genome [4]. Long noncoding RNA (lncRNA) is a novel type of ncRNAs and characherized by more than 200 nucleotides in length and the lack of protein-coding potential [5]. Emerging evidence indicated that lncRNAs act essential functions in human cancers through regulating various biological processes, such as cell growth, metastasis, stemness, and survival [6, 7]. Accumulating studies demonstrated that lncRNAs could be classified into many subgroups, such as anti-sense and long intergenic ncRNA [8]. LncRNAs exert functions via several mechanisms, such as serving as sponge and scaffold [9, 10]. Quite a number of lncRNAs are found to be aberrantly expressed in cancer. For example, lncRNA HOTTIP is upregulated in ovarian cancer and promotes tumor growth and migration [11]. LncRNA SNHG16 upregulation enhances bladder cancer cell proliferation, migration and invasion through targeting miR-98/STAT3 pathway [12]. Thus, the relationship between lncRNA and CRC progression requires to be investigated.

SOCS2-AS1 is a poorly defined lncRNA. Only one report indicated that SOCS2-AS1 contributes to prostate cancer cell proliferation [13]. This present study revealed that SOCS2-AS1 was downregulated in CRC tissues and cells. SOCS2-AS1 overexpression inhibited CRC growth and metastasis and vice versa. Our data demonstrated that SOCS2-AS1 promotes SOCS2 expression through sponging miR-1264. In conclusion, our findings discovered that SOCS2-AS1 is a novel anti-cancer lncRNA to inhibit CRC progression through regulating miR-1264/SOCS2 pathway, suggesting that SOCS2-AS1 may be a potential therapeutic target.

## RESULTS

### SOCS2-AS1 was downregulated in CRC

**Figure 1:**
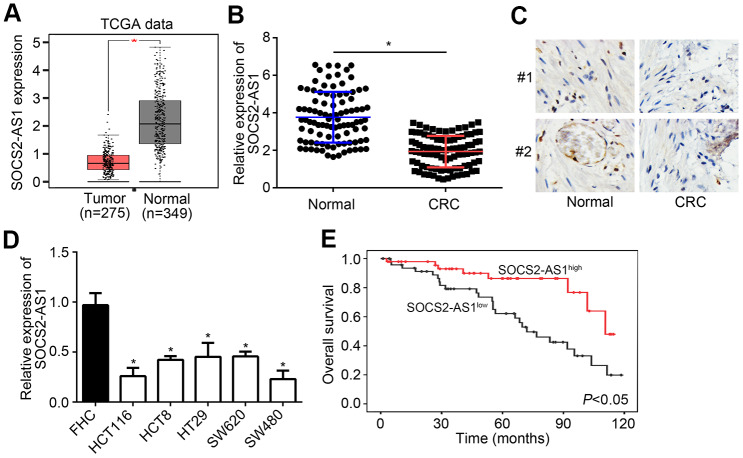
**SOCS2-AS1 was downregulated in CRC.** (**A**) Expression of SOCS2-AS1 in CRC and normal tissues according to TCGA data. GEPIA tool (http://gepia.cancer-pku.cn/detail.php) was used to analyze TCGA data. (**B**) Relative expression of SOCS2-AS1 in CRC and corresponding normal tissues was analyzed by qRT-PCR. (**C**) Analysis of SOCS2-AS1 level in CRC tissues and paired normal tissues by *in situ* hybridization (ISH). (**D**) Relative expression of SOCS2-AS1 in CRC cell lines. (**E**) Overall survival rate was plotted based on SOCS2-AS1 expression levels in CRC tissues. *P<0.05.

According to TCGA data, SOCS2-AS1 expression was observed to be downregulated in CRC tissues compared to normal tissues (Figure 1A). To confirm it, qRT-PCR was performed to analyze SOCS2-AS1 expression in CRC tissues and paired normal tissues. Results showed that SOCS2-AS1 level was decreased in CRC tissues (Figure 1B), which was validated by ISH analysis (Figure 1C). SOCS2-AS1 expression was also downregulated in CRC cell lines compared with FHC cells (Figure 1D). Moreover, it was observed that SOCS2-AS1 high expression was correlated with a high survival rate in CRC patients (Figure 1E).

### SOCS2-AS1 overexpression inhibited CRC proliferation, migration and invasion

**Figure 2:**
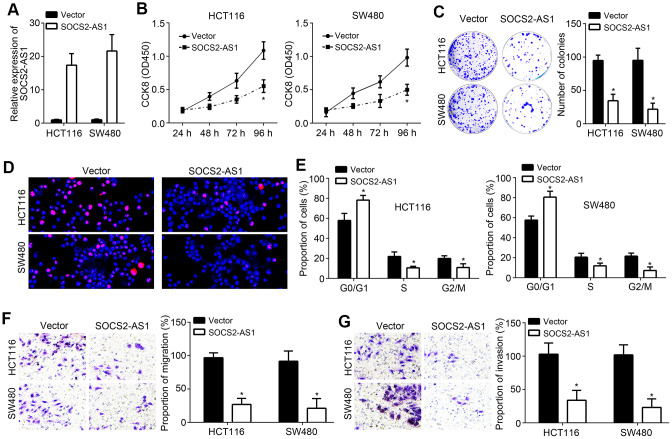
**SOCS2-AS1 overexpression inhibited CRC proliferation, migration and invasion.** (**A**) SOCS2-AS1 expression was upregulated in HCT116 and SW480 cells after transfection with SOCS2-AS1 overexpressing vector. (**B**, **C**) CCK8 and colony formation assays were performed to determine CRC cell proliferation. (**D**) Cell proliferation was evaluated by immunofluorescence staining with EdU. (**E**) Cell-cycle status of CRC cells were analyzed by FACS. (**F**, **G**) Transwell assay was performed to analyze cell migration and invasion. *P<0.05.

To explore the function of SOCS2-AS1, we generated SOCS2-AS1 overexpressed HCT116 and SW480 cell lines (Figure 2A). Through CCK8 and colony formation assays, we observed that SOCS2-AS1 overexpression inhibited proliferation and colony formation of HCT116 and SW480 cells (Figure 2B, 2C), suggesting that SOCS2-AS1 represses CRC proliferation. EdU staining further validated above conclusion (Figure 2D). Moreover, we found that SOCS2-AS1 overexpression led to more cells arrested in G0/G1 phase (Figure 2E), indicating that SOCS2-AS1 upregulation impairs cell-cycle progression. Additionally, transwell assay was performed and results showed that SOCS2-AS1 ectopic expression resulted in decreased numbers of cell migration and invasion (Figure 2F, 2G).

### SOCS2-AS1 knockdown contributed to CRC progression

**Figure 3:**
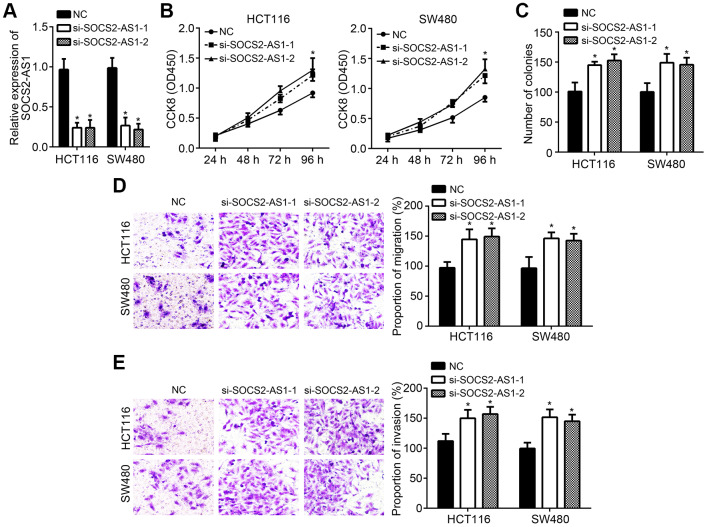
**SOCS2-AS1 knockdown contributed to CRC progression.** (**A**) SOCS2-AS1 expression was decreased after transfection with SOCS2-AS1 siRNAs. (**B**, **C**) CCK8 and colony formation assays were conducted to estimate cell proliferation. (**D**, **E**) Transwell assay was performed to analyze cell migration and invasion. *P<0.05.

To further validate the roles of SOCS2-AS1 in CRC, two siRNAs were also used to knock down SOCS2-AS1 (Figure 3A). Similarly, CCK8 and colony formation assays demonstrated that SOCS2-AS1 knockdown enhanced cell proliferation and promoted colony formation (Figure 3B, 3C). Transwell assay also indicated that more migrated and invaded cells were observed in si-SOCS2-AS1 group (Figure 3D, 3E).

### SOCS2-AS1 promoted SOCS2 expression through sponging miR-1264

**Figure 4:**
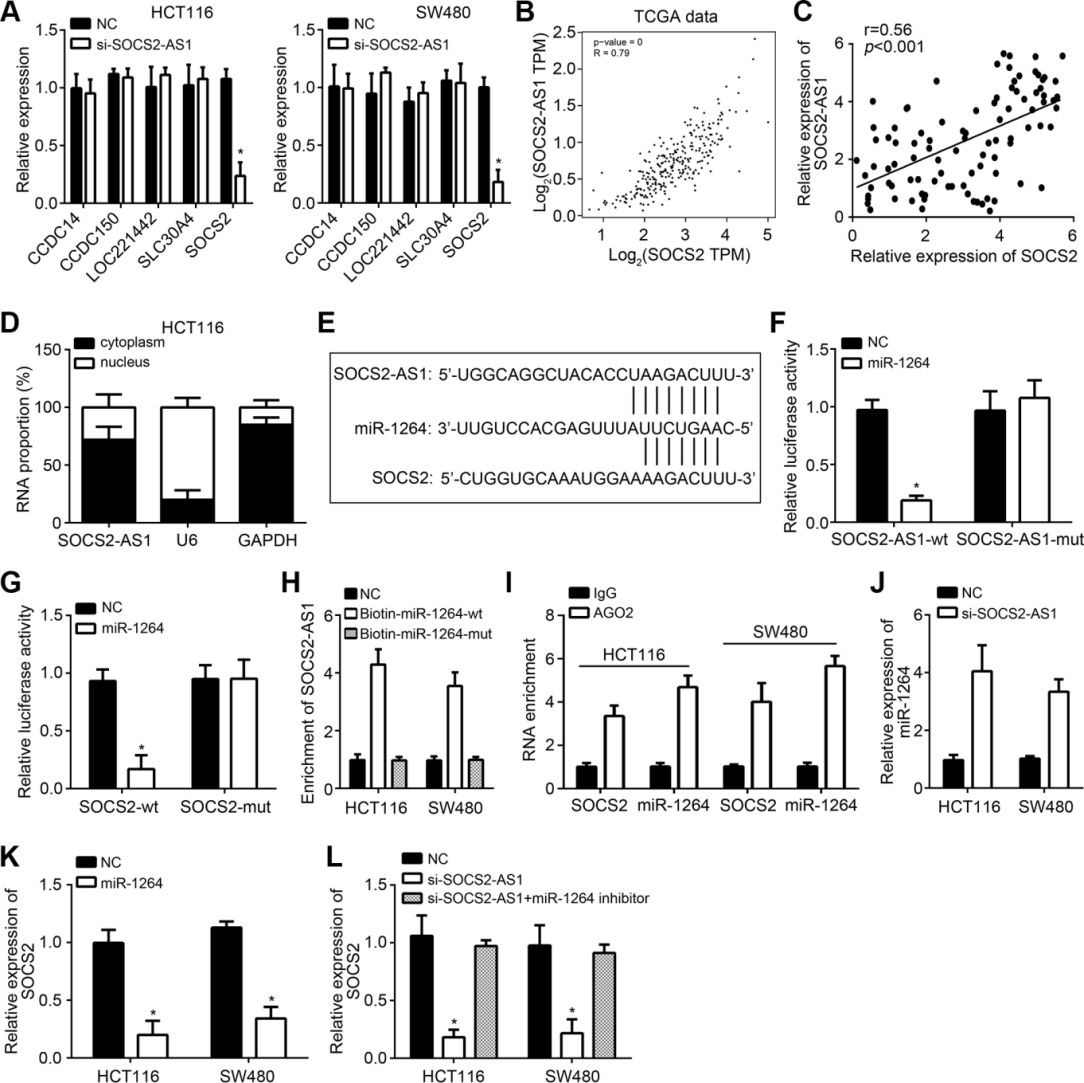
**SOCS2-AS1 promoted SOCS2 expression through sponging miR-1264.** (**A**) qRT-PCR analysis for indicated genes. (**B**) Expression correlation between SOCS2-AS1 and SOCS2 in CRC tissues according to TCGA data. GEPIA tool (http://gepia.cancer-pku.cn/detail.php) was used to analyze TCGA data. (**C**) qRT-PCR was used to expression correlation between SOCS2-AS1 and SOCS2 in CRC tissues. (**D**) Subcellular location of SOCS2-AS1 was determined by qRT-PCR. (**E**) Predicted binding sites among SOCS2-AS1, miR-1264 and SOCS2 through miRDB tool (http://mirdb.org/). (**F**, **G**) Luciferase reporter assays were carried out to validate the interactions among SOCS2-AS1, miR-1264 and SOCS2. (**H**) RNA pulldown assay was conducted to confirm the interaction between SOCS2-AS1 and miR-1264. (**I**) RIP assay was used to confirm the interaction between SOCS2 and miR-1264. (**J**) SOCS2-AS1 knockdown promoted miR-1264 level. (**K**) miR-1264 mimics inhibited SOCS2 expression. (**L**) SOCS2-AS1 knockdown inhibited SOCS2 expression through miR-1264. *P<0.05.

To investigate the functional mechanism of SOCS2-AS1, we used TANRIC tool (https://ibl.mdanderson.org/tanric/_design/basic/summary.html) and performed bioinformatics analysis to search target genes which are correlated with SOCS2-AS1 level in CRC tissues. We found out five candidates (CCDC14, CCDC150, LOC221442, SLC30A4 and SOCS2) which are positively correlated with SOCS2-AS1 expression. Nevertheless, we only observed that SOCS2-AS1 knockdown inhibited SOCS2 expression (Figure 4A), suggesting that SOCS2 may be the target of SOCS2-AS1. TCGA data using GEPIA tool also indicated that SOCS2-AS1 and SOCS2 expressions were positively correlated in CRC tissues (Figure 4B), which was validated by qRT-PCR (Figure 4C). SOCS2-AS1 was mainly expressed in the cytoplasm (Figure 4D), implying SOCS2-AS1 may be a ceRNA. Through bioinformatics analysis using miRDB, we only found that both SOCS2-AS1 and SOCS2 had the response element with miR-1264 (Figure 4E). According to the results from luciferase reporter assay, we found that miR-1264 mimics suppressed the activity of SOCS2-AS1-wt and SOCS2-wt (Figure 4F, 4G), indicating miR-1264 interacted with SOCS2-AS1 and SOCS2. RNA pulldown and RIP assays further validated the results of luciferase reporter assay (Figure 4H, 4I). Additionally, we found that miR-1264 level was increased after SOCS2-AS1 knockdown (Figure 4J). And SOCS2 expression was suppressed by miR-1264 mimics (Figure 4K). Furthermore, we observed that miR-1264 inhibitors reversed the effects of SOCS2-AS1 knockdown on SOCS2 expression (Figure 4L), indicating that SOCS2-AS1 promotes SOCS2 expression through sponging miR-1264.

### SOCS2-AS1 suppressed CRC progression through promoting SOCS2 expression

**Figure 5:**
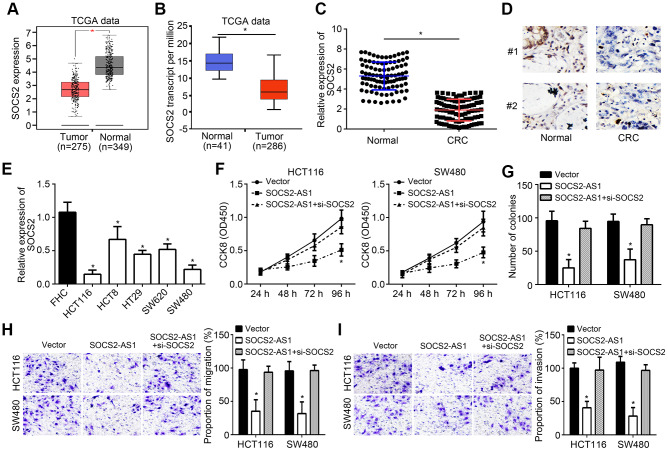
**SOCS2-AS1 suppressed CRC progression through promoting SOCS2 expression.** (**A**) Expression of SOCS2 in TCGA data through GEPIA tool (http://gepia.cancer-pku.cn/detail.php). (**B**) Expression of SOCS2 in TCGA data through UALCAN tool (http://ualcan.path.uab.edu/analysis.html). (**C**) qRT-PCR analysis of SOCS2 expression in CRC tissues and normal tissues. (**D**) IHC analysis for SOCS2 expression in CRC tissues and paired normal tissues. (**E**) Expression levels of SOCS2 were determined in CRC cell lines by qRT-PCR. (**F**, **G**) CCK8 and colony formation assays were utilized to estimate cell proliferation. (**H**, **I**) Transwell assay was conducted to test cell migration and invasion. *P<0.05.

TCGA data from GEPIA and UALCAN tools indicated that SOCS2 was downregulated in CRC tissues compared to normal tissues (Figure 5A, 5B), which was demonstrated by qRT-PCR (Figure 5C). IHC staining also suggested that SOCS2 was lowly expressed in CRC tissues (Figure 5D). Consistently, SOCS2 was downregulated in CRC cell lines (Figure 5E). To investigate whether SOCS2-AS1 affects CRC progression through SOCS2, we carried out CCK8, colony formation and transwell assays. Results indicated that SOCS2 knockdown reversed the suppressive roles of SOCS2-AS1 overexpression on CRC proliferation and metastasis (Figure 5F–5I).

### *In vivo* effects of SOCS2-AS1 overexpression on CRC growth and metastasis

**Figure 6:**
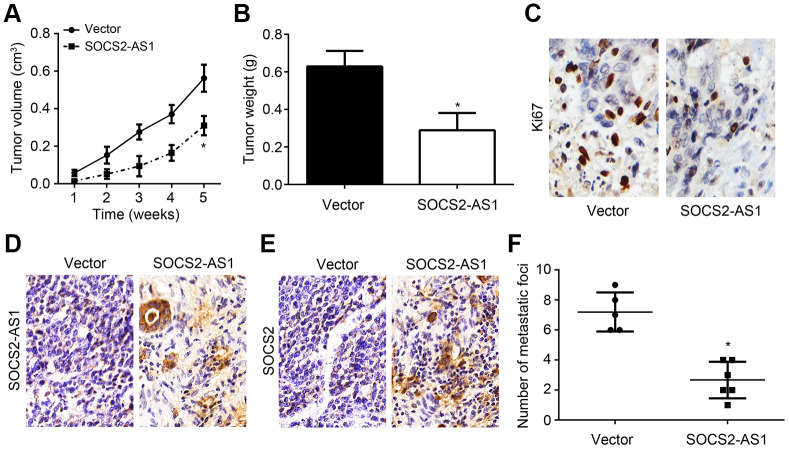
***In vivo* effects of SOCS2-AS1 overexpression on CRC growth and metastasis.** (**A**) Tumor volumes were measured every week. (**B**) Tumor weights were determined after 5 weeks. (**C**) Ki67 expression was analyzed by IHC in tumor tissues. (**D**) Expression of SOCS2-AS1 in tumor tissues was measured by ISH. (**E**) Expression of SOCS2 was evaluated by IHC. (**F**) Number of metastatic foci in liver was analyzed. *P<0.05.

To further reveal the roles of SOCS2-AS1 *in vivo*, xenograft assay and metastasis assay were carried out. As shown, SOCS2-AS1 overexpression inhibited tumor volumes and weights (Figure 6A, 6B). And the Ki67 positive tumor cells were fewer in si-SOCS2-AS1 group (Figure 6C). Obviously, SOCS2-AS1 and SOCS2 expressions were still higher in tumor tissues of si-SOCS2-AS1 group (Figure 6D, 6E). Numbers of metastatic tumor tissues in liver of si-SOCS2-AS1 group was decreased compared to that in NC group (Figure 6F). Thus, SOCS2-AS1 overexpression suppresses CRC growth and metastasis *in vivo*.

## DISCUSSION

How lncRNAs regulate CRC progression still remains undefined. In this study, we identified that lncRNA SOCS2-AS1 was lowly expressed in CRC tissues and cell lines. Moreover, SOCS2-AS1 low expression was related with a low survival rate in CRC patients. Gain-of-function assays and loss-of-function assays demonstrated that SOCS2-AS1 inhibited CRC cell proliferation, migration and invasion *in vitro* and *in vivo*. In terms of mechanism, SOCS2-AS1 was found to facilitate SOCS2 expression through acting as a ceRNA for miR-1264. The present work illustrated the novel function and mechanism of SOCS2-AS1 in CRC progression.

Mounting studies have showed that lncRNAs play critical roles in cancer pathology and may be potential targets for diagnosis and prognosis [14]. Several lncRNAs are reported to regulate CRC progression. For example, LINC00483 contributes to CRC growth and invasion through affecting FMNL2 expression [15]. Lnc-kcna3 upregulation suppresses CRC cell proliferation, migration and invasion through negatively regulating YAP1 level [16]. In addition, lncRNA PiHL affects CRC progression through regulating P53 stability [17]. SOCS2-AS1 was proven to enhance prostate cancer growth [13]. Nevertheless, the function and mechanism of SOCS2-AS1 in CRC is unclear. In our study, we found that SOCS2-AS1 expression was downregulated in CRC tissues through TCGA data and qRT-PCR analysis. And high SOCS2-AS1 expression was associated with high survival rate. Moreover, through CCK8, colony formation, EdU staining and transwell assays, we demonstrated that SOCS2-AS1 inhibited CRC proliferation, migration and invasion *in vitro*. Moreover, SOCS2-AS1 upregulation repressed CRC growth and liver metastasis *in vivo*. Our findings demonstrated that SOCS2-AS1 acts as a new tumor suppressor in CRC.

Growing numbers of works have indicated that lncRNAs are involved in ceRNAs by sponging miRNAs [9, 12]. For instance, HULC overexpression promotes liver cancer development and invasion via sponging miR-372 [18]. LncRNA H19 contributes to breast cancer cell growth and stemness through modulating let-7 [19]. Additionally, lncRNA RP4 acts as a ceRNA for miR-7-5p to regulate CRC progression [20]. At present, whether SOCS2-AS1 could work as a ceRNA remains unknown. Our results suggested that SOCS2-AS1 was expressed in the cytoplasm of CRC cells. After bioinformatics analysis, we found that SOCS2-AS1 may target miR-1264. We demonstrated the direct interaction between miR-1264 and SOCS2-AS1 through luciferase reporter assay and RNA pulldown assay. MiR-1264 is a functionally unclear miRNA. Our study for the first time suggested that miR-1264 was regulated by SOCS2-AS1 and contributed to CRC progression.

We also found that SOCS2 may be a target of miR-1264. Through luciferase reporter assay and RIP assay, we demonstrated the relationship between SOCS2 and miR-1264. SOCS2, a suppressor of cytokine signaling, has been reported to be tumor suppressor in various cancers. For example, SOCS2 downregulation promotes prostate cancer proliferation and metastasis [21]. SOCS2 is a tumor suppressor to inhibit proliferation and invasion of human laryngeal squamous cell cancer [22]. Additionally, downregulation of SOCS2 leads to enhanced proliferation and reduced apoptosis in lung cancer [23]. However, how SOCS2 affects CRC progression remains uninvestigated. In our study, we found that SOCS2-AS1 was positively correlated with SOCS2 level in CRC tissues. Moreover, we found that SOCS2 expression was inhibited by miR-1264. Additionally, we demonstrated that SOCS2-AS1 facilitates SOCS2 expression through acting as the ceRNA for miR-1264. Finally, we showed that inhibition of SOCS2 rescued the growth and metastasis of CRC cells. Therefore, SOCS2 is a tumor suppressor in CRC.

Taken together, our findings provided the first evidence that SOCS2-AS1 promotes CRC progression through facilitating SOCS2 expression by sponging miR-1264. And our results also suggested that SOCS2-AS1 may be a novel therapeutic target.

## MATERIALS AND METHODS

### Patients’ tissues

95 CRC samples and their adjacent normal tissues were obtained from The First Affiliated Hospital of Wenzhou Medical University. All tissues were stored in liquid nitrogen until used. The study was approved by the ethics committee of The First Affiliated Hospital of Wenzhou Medical University. And written informed consent was collected from each patient.

### Cell culture and transfection

All human CRC cell lines and human normal colorectal epithelial cell line FHC were obtained from the American Type Culture Collection. Cells were cultured using Dulbecco’s modified Eagle’s medium (DMEM) with 10% fetal bovine serum. SOCS2-AS1 full-length was inserted into the expression vector pLenti-Glll-GMV-GFP-2A-Puro (Applied Biological Materials, Canada) for SOCS2-AS1 overexpression. Small interfering RNAs (siRNAs) targeting SOCS2-AS1 (#1: 5’-UUAUCACUCAUCAUUUCAGAA-3’; #2: 5’-GACCUGUAUGGUCAUUAUCACUC-3’) or SOCS2 (5’-UAUAUUCUUCCAAGUAAUCUU-3’), miR-1264 mimics, miR-1264 inhibitors and negative controls were obtained from GenePharma (China). These plasmids were transfected into cells using Lipofectamine 2000 (Invitrogen, USA) according to the manufacturer’s instructions.

### qRT-PCR

Total RNA was isolated using Trizol reagent following the manufacturer’s instructions. cDNA was synthesized by using the High-Capacity cDNA Reverse Transcription Kit (Applied Biosystems, Foster City, CA, USA). qPCR was carried out through SYBR Green Real-Time PCR MasterMix (Toyobo, Osaka, Japan). GAPDH was the normalized control. Relative expression was determined according to the 2^−ΔΔCT^ method. Primer sequences were as follows: SOCS2-AS1 (Forward: 5’-CCATACAGGTCAACTTTTCCACCAC-3’ and reverse: 5’-CCAACCTCAGCTCTGCTCTCTT-3’), SOCS2 (Forward: 5’-AACCGCTCTACACGTCAGCA-3’ and reverse: 5’-TGGTAAAGGCAGTCCCCAGA-3’) and GAPDH (Forward: 5’-GGTGGTCTCCTCTGACTTCAACA-3’ and reverse: 5’-GTGGTCGTTGAGGGCAATC-3’).

### CCK8 assay

CCK8 assay was conducted to analyze proliferation. 1000 cells were seeded into 96-well plates and cultured for described hours. Then CCK8 solution was added and incubated for 2 hours. The absorbance at 450 nm was determined for each sample.

### Transwell assay

Cell migration and invasion were evaluated through transwell assay. In brief, cells were seeded into the upper chamber (only pre-coated by Matrigel for invasion assay) with 200 μl serum-free medium. The lower chamber was inoculated with 600 μl medium containing 10% FBS. Cells were cultured for 24 hours. Then migrated or invaded cells were fixed with methanol and stained with 0.1% crystal violet. TCell numbers were determined using a light inverted microscope.

### Luciferase reporter assay

The SOCS2-AS1 or SOCS2 sequence containing wild-type (WT) or mutant (Mut) response element with miR-1264 was inserted into pmirGLO Vector (Promega, Madison, WI, USA) to generate pmirGLO-SOCS2-AS1-WT, pmirGLO-SOCS2-AS1-Mut, pmirGLO-SOCS2–WT and pmirGLO-SOCS2-Mut vectors. Then luciferase vectors and miR-1264 mimics were transfected into HCT116 cells using Lipofectamine 2000 and cultured for 48 hours. The relative luciferase activity was determined through a Dual-Luciferase Reporter Assay Kit (Promega).

### Xenograft assay and *in vivo* metastasis assay

5-week-old BALB/c-nude mice were randomly divided into two groups. For xenograft assay, HCT116 cells (5 × 10^6^ cells) were subcutaneously implanted in right flank of BALB/c-nu mice (n=5 per group). Tumor volumes were measured every week. And tumor weights were determined after 5 weeks. Tumor tissues were subjected to paraffin embedding and used for hematoxylin and eosin (H&E) staining. For liver metastasis *in vivo*, HCT116 cells (3 × 10^6^/0.2 ml PBS) were injected into nude mice through the tail vein. Two months later, the lungs were isolated and metastasis was analyzed. All animal experiments were approved by the ethics committee of The First Affiliated Hospital of Wenzhou Medical University.

### RNA immunoprecipitation assay (RIP)

RIP assay was carried out using An EZ Magna RNA immunoprecipitation Kit (Millipore, USA) based on the manufacturer’s guidelines. Briefly, cells were lysed through RIP lysis buffer and incubated with anti-AGO2 or IgG for 2 h and further incubated with Magnetic beads for another 2 h. Then immunoprecipitated RNA was extracted and analyzed by qRT-PCR.

### Statistical analysis

All analyses were performed using GraphPad Prism 6 (GraphPad, USA) software and results were expressed as the mean ± standard deviation (SD). Student’s t test or one-way ANOVA was used for analyses of statistical differences. Kaplan–Meier method and the log-rank test were used to analyze overall survival rate. P < 0.05 was considered statistically significant.

## References

[1] Brenner H, Kloor M, Pox CP. Colorectal cancer. Lancet. 2014; 383:1490–502. 10.1016/S0140-6736(13)61649-9 24225001

[2] Sadanandam A, Lyssiotis CA, Homicsko K, Collisson EA, Gibb WJ, Wullschleger S, Ostos LC, Lannon WA, Grotzinger C, Del Rio M, Lhermitte B, Olshen AB, Wiedenmann B, et al. A colorectal cancer classification system that associates cellular phenotype and responses to therapy. Nat Med. 2013; 19:619–25. 10.1038/nm.3175 23584089 PMC3774607

[3] Nishihara R, Wu K, Lochhead P, Morikawa T, Liao X, Qian ZR, Inamura K, Kim SA, Kuchiba A, Yamauchi M, Imamura Y, Willett WC, Rosner BA, et al. Long-term colorectal-cancer incidence and mortality after lower endoscopy. N Engl J Med. 2013; 369:1095–105. 10.1056/NEJMoa1301969 24047059 PMC3840160

[4] Esteller M. Non-coding RNAs in human disease. Nat Rev Genet. 2011; 12:861–74. 10.1038/nrg3074 22094949

[5] Wang KC, Chang HY. Molecular mechanisms of long noncoding RNAs. Mol Cell. 2011; 43:904–14. 10.1016/j.molcel.2011.08.018 21925379 PMC3199020

[6] Khaitan D, Dinger ME, Mazar J, Crawford J, Smith MA, Mattick JS, Perera RJ. The melanoma-upregulated long noncoding RNA SPRY4-IT1 modulates apoptosis and invasion. Cancer Res. 2011; 71:3852–62. 10.1158/0008-5472.CAN-10-4460 21558391

[7] Ma Y, Yang Y, Wang F, Moyer MP, Wei Q, Zhang P, Yang Z, Liu W, Zhang H, Chen N, Wang H, Wang H, Qin H. Long non-coding RNA CCAL regulates colorectal cancer progression by activating Wnt/β-catenin signalling pathway via suppression of activator protein 2α. Gut. 2016; 65:1494–504. 10.1136/gutjnl-2014-308392 25994219

[8] Chen DL, Chen LZ, Lu YX, Zhang DS, Zeng ZL, Pan ZZ, Huang P, Wang FH, Li YH, Ju HQ, Xu RH. Long noncoding RNA XIST expedites metastasis and modulates epithelial-mesenchymal transition in colorectal cancer. Cell Death Dis. 2017; 8:e3011. 10.1038/cddis.2017.421 28837144 PMC5596599

[9] Chen DL, Ju HQ, Lu YX, Chen LZ, Zeng ZL, Zhang DS, Luo HY, Wang F, Qiu MZ, Wang DS, Xu DZ, Zhou ZW, Pelicano H, et al. Long non-coding RNA XIST regulates gastric cancer progression by acting as a molecular sponge of miR-101 to modulate EZH2 expression. J Exp Clin Cancer Res. 2016; 35:142. 10.1186/s13046-016-0420-1 27620004 PMC5020507

[10] Liu B, Ye B, Yang L, Zhu X, Huang G, Zhu P, Du Y, Wu J, Qin X, Chen R, Tian Y, Fan Z. Long noncoding RNA lncKdm2b is required for ILC3 maintenance by initiation of Zfp292 expression. Nat Immunol. 2017; 18:499–508. 10.1038/ni.3712 28319097

[11] Zou T, Wang PL, Gao Y, Liang WT. Long noncoding RNA HOTTIP is a significant indicator of ovarian cancer prognosis and enhances cell proliferation and invasion. Cancer Biomark. 2019; 25:133–39. 10.3233/CBM-181727 30452402 PMC13082406

[12] Feng F, Chen A, Huang J, Xia Q, Chen Y, Jin X. Long noncoding RNA SNHG16 contributes to the development of bladder cancer via regulating miR-98/STAT3/wnt/β-catenin pathway axis. J Cell Biochem. 2018; 119:9408–18. 10.1002/jcb.27257 30132983

[13] Misawa A, Takayama K, Urano T, Inoue S. Androgen-induced long noncoding RNA (lncRNA) SOCS2-AS1 promotes cell growth and inhibits apoptosis in prostate cancer cells. J Biol Chem. 2016; 291:17861–80. 10.1074/jbc.M116.718536 27342777 PMC5016176

[14] Jing H, Qu X, Liu L, Xia H. A novel long noncoding RNA (lncRNA), LL22NC03-N64E9.1, promotes the proliferation of lung cancer cells and is a potential prognostic molecular biomarker for lung cancer. Med Sci Monit. 2018; 24:4317–23. 10.12659/MSM.908359 29935018 PMC6047588

[15] Yan Y, Wang Z, Qin B. A novel long noncoding RNA, LINC00483 promotes proliferation and metastasis via modulating of FMNL2 in CRC. Biochem Biophys Res Commun. 2019; 509:441–47. 10.1016/j.bbrc.2018.12.090 30594388

[16] Zhong X, Lü M, Wan J, Zhou T, Qin B. Long noncoding RNA kcna3 inhibits the progression of colorectal carcinoma through down-regulating YAP1 expression. Biomed Pharmacother. 2018; 107:382–89. 10.1016/j.biopha.2018.07.118 30099342

[17] Deng X, Li S, Kong F, Ruan H, Xu X, Zhang X, Wu Z, Zhang L, Xu Y, Yuan H, Peng H, Yang D, Guan M. Long noncoding RNA PiHL regulates p53 protein stability through GRWD1/RPL11/MDM2 axis in colorectal cancer. Theranostics. 2020; 10:265–80. 10.7150/thno.36045 31903119 PMC6929633

[18] Wang J, Liu X, Wu H, Ni P, Gu Z, Qiao Y, Chen N, Sun F, Fan Q. CREB up-regulates long non-coding RNA, HULC expression through interaction with microRNA-372 in liver cancer. Nucleic Acids Res. 2010; 38:5366–83. 10.1093/nar/gkq285 20423907 PMC2938198

[19] Peng F, Li TT, Wang KL, Xiao GQ, Wang JH, Zhao HD, Kang ZJ, Fan WJ, Zhu LL, Li M, Cui B, Zheng FM, Wang HJ, et al. H19/let-7/LIN28 reciprocal negative regulatory circuit promotes breast cancer stem cell maintenance. Cell Death Dis. 2017; 8:e2569. 10.1038/cddis.2016.438 28102845 PMC5386357

[20] Liu ML, Zhang Q, Yuan X, Jin L, Wang LL, Fang TT, Wang WB. Long noncoding RNA RP4 functions as a competing endogenous RNA through miR-7-5p sponge activity in colorectal cancer. World J Gastroenterol. 2018; 24:1004–12. 10.3748/wjg.v24.i9.1004 29531464 PMC5840465

[21] Das R, Gregory PA, Fernandes RC, Denis I, Wang Q, Townley SL, Zhao SG, Hanson AR, Pickering MA, Armstrong HK, Lokman NA, Ebrahimie E, Davicioni E, et al. MicroRNA-194 promotes prostate cancer metastasis by inhibiting SOCS2. Cancer Res. 2017; 77:1021–34. 10.1158/0008-5472.CAN-16-2529 28011622

[22] Zhao X, Zhang W, Ji W. miR-196b is a prognostic factor of human laryngeal squamous cell carcinoma and promotes tumor progression by targeting SOCS2. Biochem Biophys Res Commun. 2018; 501:584–92. 10.1016/j.bbrc.2018.05.052 29753737

[23] Tong JL, Wang LL, Ling XF, Wang MX, Cao W, Liu YY. MiR-875 can regulate the proliferation and apoptosis of non-small cell lung cancer cells via targeting SOCS2. Eur Rev Med Pharmacol Sci. 2019; 23:5235–41. 10.26355/eurrev_201906_18189 31298374

